# Molecular characterization and re-interpretation of *HNF1A* variants identified in Indian MODY subjects towards precision medicine

**DOI:** 10.3389/fendo.2023.1177268

**Published:** 2023-06-16

**Authors:** Babu Kavitha, Sampathkumar Ranganathan, Sundaramoorthy Gopi, Umashankar Vetrivel, Nagarajan Hemavathy, Viswanathan Mohan, Venkatesan Radha

**Affiliations:** ^1^ Department of Molecular Genetics, Madras Diabetes Research Foundation, Indian Council of Medical Research (ICMR) Centre for Advanced Research on Diabetes, Affiliated to University of Madras, Chennai, India; ^2^ Centre for Bioinformatics, School of Life Sciences, Pondicherry University, Puducherry, India; ^3^ Department of Bioinformatics, Vision Research Foundation, Chennai, India; ^4^ Department of Virology Biotechnology, Indian Council of Medical Research (ICMR)-National Institute of Traditional Medicine, Belagavi, India; ^5^ Department of Diabetology, Madras Diabetes Research Foundation, Chennai and Dr. Mohan’s Diabetes Specialties Centre, International Diabetes Federation (IDF) Centre of Education, Chennai, India

**Keywords:** Maturity Onset Diabetes of Young (MODY) subtype-3, acmg-amp guidelines, re-interpretation, pathogenic variants, functional characterization, structural analysis, ACMG-AMP guidelines

## Abstract

**Background:**

*HNF1A* is an essential component of the transcription factor network that controls pancreatic β-cell differentiation, maintenance, and glucose stimulated insulin secretion (GSIS). A continuum of protein malfunction is caused by variations in the *HNF1A* gene, from severe loss-of-function (LOF) variants that cause the highly penetrant Maturity Onset Diabetes of the Young (MODY) to milder LOF variants that are far less penetrant but impart a population-wide risk of type 2 diabetes that is up to five times higher. Before classifying and reporting the discovered variations as relevant in clinical diagnosis, a critical review is required. Functional investigations offer substantial support for classifying a variant as pathogenic, or otherwise as advised by the American College of Medical Genetics and Genomics (ACMG) and the Association for Molecular Pathology (AMP) ACMG/AMP criteria for variant interpretation.

**Objective:**

To determine the molecular basis for the variations in the *HNF1A* gene found in patients with monogenic diabetes in India.

**Methods:**

We performed functional protein analyses such as transactivation, protein expression, DNA binding, nuclear localization, and glucose stimulated insulin secretion (GSIS) assay, along with structural prediction analysis for 14 *HNF1A* variants found in 20 patients with monogenic diabetes.

**Results:**

Of the 14 variants, 4 (28.6%) were interpreted as pathogenic, 6 (42.8%) as likely pathogenic, 3 (21.4%) as variants of uncertain significance, and 1 (7.14%) as benign. Patients harboring the pathogenic/likely pathogenic variants were able to successfully switch from insulin to sulfonylureas (SU) making these variants clinically actionable.

**Conclusion:**

Our findings are the first to show the need of using additive scores during molecular characterization for accurate pathogenicity evaluations of *HNF1A* variants in precision medicine.

## 1 Introduction

The hepatocyte nuclear factor 1A **(*HNF1A*)**gene (MIM # 142410) encodes a crucial member of an auto-regulatory transcription circuit in mature and developing pancreas. Heterozygous mutations in *HNF1A* result in the most common form of MODY namely subtype *HNF1A*-MODY. Autosomal dominant inheritance, early onset, and progressive β-cell deterioration resulting in severe hyperglycemia define this type of monogenic diabetes (1–3). This kind of MODY has the highest prevalence and is more common than other subtypes, and it is more common in Europe, North America, and Asia (4–7).

Individuals with *HNF1A* MODY are likely to develop extra pancreatic symptoms such as glycosuria which will appear even before the onset of diabetes due to a low renal glucose threshold (8). This is mainly because *HNF1A* is expressed in tissues such as the kidney, liver, and small intestine, in addition to β-cells. The risk of micro- and macro-vascular problems in *HNF1A*-MODY is comparable to that of T1D and T2DM (9) and hence strict glucose management is required for these individuals. Patients harboring pathogenic variants in *HNF1A* gene are sensitive to low doses of sulfonylureas (10).

The *HNF1A* protein consists of three functional domains namely a dimerization domain (1 – 33 aa), a bipartite DNA-binding domain (homeo domain 100 –184 aa; POU domain 198 –281 aa), and a transactivation domain (282 –631 aa) (11, 12). It binds to DNA as a homodimer or with the structurally related transcription factor *HNF1B* as heterodimers (13, 14). To date, about 564 MODY-causing variants have been identified in the *HNF1A* gene (15, 16). These variations include missense, nonsense, frameshift, in-frame deletions/insertions/duplications, splice site, promoter region, and whole/partial gene deletions. Analyses of these variants have demonstrated that some of them render the protein unstable and poorly expressed (17, 18). Some of the variants affect either the DNA binding or transactivation ability of *HNF1A*. However, patients with the latter type of variants do not exhibit more severe phenotypes (19–21). Finally, a subgroup of variants exert a dominant-negative effect over the normal protein.

It is important that these candidate variants are subjected to rigorous evaluation of pathogenicity to avoid false annotation of causality, which would be an impediment to the translation of genomic research findings to clinical practice and precision medicine. False assignment of pathogenicity can also have severe consequences for patients, resulting in incorrect prognostic and therapeutic advice. Therefore, a comprehensive map is needed, linking mutation status, effect on protein function, and clinical effect that is genotype-function-phenotype. The recent American College of Medical Genetics and Genomics (ACMG) and the Association for Molecular Pathology (AMP) (ACMG-AMP) guidelines classification is based on five tier score system namely pathogenic (P), likely pathogenic (LP), variant of uncertain significance (VUS), likely benign (LB) and benign (B) (22). Our previous studies have shown that *HNF1A* -MODY is the most prevalent subtype in India (3) and we identified several variants which were of uncertain significance, Assessing the pathogenicity of these rare protein-coding genetic variants in *HNF1A* is very important in our patient cohort before assigning causality to these variants, as this may lead to change of treatment.

Functional investigation constitutes one of the strongest pieces of evidence for classifying a variant as pathogenic or benign (23). Each variant needs to be assessed by genomic, bioinformatic, structural, and functional lines of evidence for classifying them as pathogenic or benign. Hence, we hypothesized that functional evaluation would enhance the interpretation of the pathogenicity of *HNF1A* variants identified in individuals from families of Indian MODY subjects.

## 2 Materials and methods

### 2.1 Subjects

We investigated 14 *HNF1A* variants found in 20 unrelated individuals (11 females and 9 males) from 20 non-consanguineous Indian families. Patients were selected for MODY genetic screening based on the following criteria: a family history of diabetes in multiple generations; an early age at onset of diabetes (< 35 years); lack of obesity, ketosis, and beta cell autoimmunity with detectable endogenous insulin reserve as measured by C peptide which is one of the best biomarkers; and diabetes controllable without insulin for at least 2 years. The study was carried out in compliance with the Helsinki Declaration (2000); all study participants (or their guardians) provided written, informed consent, and the study was approved by the Madras Diabetes Research Foundation’s local institutional ethics committee.

### 2.2 Genomic analyses

Genomic DNA was isolated from whole blood using the standard protocol. Direct sequencing was carried out on an ABI 3500 Genetic Analyzer (Applied Biosystems, Foster City, CA) using the Big Dye terminator V3.1 chemistry, and the sequences were compared with the public databases. Published primer sequences were used to amplify the DNA for *HNF1A* gene. In addition to the sequencing of patients, we also sequenced 100 normal glucose-tolerant subjects (fasting value <100 mg/dL and 2 hours value <140 mg/dL) to check for the presence or absence of variants in them.

### 2.3 ACMG classification

All *HNF1A* variations were assessed using the ACMG guidelines, which classify variants as pathogenic (class 5), likely pathogenic (class 4), uncertain significance (class 3), likely benign (class 2), or benign (class 1). Criteria used for the classification of variants are listed in **Supplementary Table 1**. Public databases such as PubMed, the Human Gene Mutation Database, ClinVar, and LOVD were used and the genome aggregation database (GnomAD) was referred to for population frequency. Bioinformatic prediction tools such as SIFT, PolyPhen2, Mutation Taster, PROVEAN, CADD Score, i mutant 2.0, and Grantham scores were used to assess the pathogenicity (**Supplementary Table 2**).

### 2.4 Functional analysis

Human *HNF1A* cDNA (NCBI Entrez Gene BC104910.1) (NM_000545.5) in pcDNA 3.1 His/C vector (Invitrogen Inc, Carlsbad, CA, USA), was used as a template for constructing individual *HNF1A* variants using the QuikChange Lightning Site-directed Mutagenesis Kit (Agilent Technologies, Santa Clara, CA), and all constructs were verified by Sanger sequencing. Transiently transfected HeLa and INS1 cells with WT, empty vector (pcDNA3.1), or variant *HNF1A* cDNA were used in functional studies, investigating *HNF1A* (i) transcriptional activity using a rat albumin (in HeLa cells) and *HNF4A P2* (in INS1 cells) promoter-linked luciferase reporter assay system; (ii) DNA binding ability was analyzed using Episeeker DNA-protein binding assay kit (Abcam, ab117139) and a biotinylated oligonucleotide (Sigma Aldrich, St. Luis, MO, US) containing the *HNF1A* binding site in the rat albumin promoter; (iii) protein expression in whole cell lysates by immunoblotting;(iv) nuclear localization by indirect immunocytochemistry; and (v) the glucose-stimulated insulin secretion (GSIS) capacity of the variant *HNF1A* in INS1 β-cells were measured using insulin ELISA kit (Mercodia, Sweden). A detailed methodology is described in the **Supplementary Material**.

### 2.5 Structural analysis

The human *HNF1A* protein sequence (P20823) was downloaded from the UniProt database. The Consurf server was used to obtain amino acid conservation scores within the orthologous protein family by comparing 150 homologous sequences. For the structure-based stability prediction, the available crystal structure of *HNF1A* in complex with DNA, PDB ID-1IC8 was remodeled with missing residues and was refined using Modeller10v. The refined Wild type (WT) *HNF1A* was considered for stability analysis of *HNF1A* and also the impact of mutants in the *HNF1A*-DNA complex. The structure of mutants was modeled with a WT-*HNF1A* template using Modeller10v, and the refined WT and MT *HNF1A* were subjected to molecular dynamics simulation studies using Gromacs2020 (10.1080/07391102.2021.1965030). Subsequently, PCA and FEL analyses were carried out to determine the near-native conformation, wherein the *HNF1A*-DNA interactions were analyzed using DNAproDB. A detailed methodology is given in the **Supplementary Material**.

### 2.6 Statistical analysis

The results of functional analyses of individual variants are presented as mean (in %) ± standard deviation (SD) and normalized to WT *HNF1A* activity (set as 100%), unless otherwise specified. Experiments were carried out on at least 3 independent occasions unless otherwise specified in the figure legends. Statistical differences between individual variants and WT function were analyzed using GraphPad Prism software (version 8.1.1, GraphPad Software, Inc. San Diego, CA, USA) and raw data (i.e., firefly/renilla ratios) and an unpaired 2-tailed t-test based on n=3. A p-value < 0.05 was considered statistically significant.

## 3 Results

### 3.1 Clinical and biochemical characteristics of the subjects with *HNF1A* variants

A total of 14 missense *HNF1A* variants identified in 20 clinical MODY patients were included in this study. All the patients were heterozygous for the variants. In three families, we were able to observe the segregation of variants in affected family members, but for other patients, family samples were not available. Pedigrees of the available families are shown in **Supplementary Figure 1**. All were negative for β-cell autoantibodies such as GAD and ZnT8 antibodies. The mean ± SD of biochemical parameters were as follows: age at onset of diabetes, 21 ± 6.5 years; Body Mass Index (BMI) - 23 ± 4 kg/m2; duration of diabetes, 9.9 ± 6.7 years; Fasting plasma glucose - 181 ± 64 mg/dL; post prandial plasma glucose - 277 ± 97 mg/dL; glycated hemoglobin (HbA1C)- 9.2 ± 2.4%; fasting C-peptide was 0.9 ± 0.4 pmol/L; stimulated C- peptide was 1.5 ± 0.6 pmol/L; total cholesterol - 169 ± 41 mg/dL; triglycerides - 137 ± 82 mg/dL; High Density Lipoprotein (HDL)- cholesterol - 39 ± 8.5 mg/dL and Low Density Lipoprotein (LDL)- cholesterol - 94 ± 36 mg/dL. Prior to functional genetic investigations, 11 patients were on insulin treatment; one patient was on insulin + metformin; four patients were on insulin + SU; one patient was on metformin alone and three patients were on SU treatment alone before the genetic investigation. Clinical and biochemical parameters are summarized in **Table 1**.

**Table 1:** Clinical and biochemical workup of subjects with *HNF1A* gene variants.

S. No	Patient ID	Gender	Variant	Age at onset (Years)	Duration of Diabetes (Years)	BMI (Kg/m2)	Fasting plasma glucose (mg/dl)	Post prandial plasma glucose (mg/dl)	HbA1C (%)	Fasting C-peptide (pmol/l)	Stimulated-C-peptide (pmol/l)	Total cholesterol (mg/dl)	Triglycerides (mg/dl)	HDL (mg/dl)	LDL (mg/dl)
1	M-026	F	p.Lys120Asn	14	3.7	19.1	188	315	7.1	0.7	1.1	127	61	33	82
2	M-027	M	p.Gln125His	26	6.3	24	134	248	6.9	1	2.2	150	167	32	85
3	M-028	F	p.Asn127Del	14.9	18.1	19.1	277	414	9.5	0.6	0.8	177	134	47	101
4	M-124	M	p.Val134Ile	26.7	6.3	21.9	194	390	9.8	0.5	0.8	136	174	27	94
5	M-125	M	p.Arg200Trp	22.8	16.1	17.9	161	280	8.3	0.5	1.2	152	84	47	88
6	M-126	F	p.Arg200Trp	11	1	23.2	114	171	–	0.9	–	-	-	-	-
7	M-129	F	p.Arg272His	26	8	26.9	106	204	6.4	1.2	2	250	71	45	49
8	M-130	F	p.Arg272His	23	5	23	125	220	6.9	1	2.3	191	209	28	121
9	M-131	F	p.Gly292fs*25	19.1	13	17.3	204	197	10.8	1.1	2	211	176	44	132
10	M-035	F	p.Gly292fs*25	11	4	18.6	127	225	8.7	0.9	1.5	153	114	59	98
11	M-132	M	p.Ala301Thr	28	19	-	114	155	7.3	–	–	193	136	47	125
12	M-133	M	p.Thr354Met	24.8	5	16.2	159	243	6.9	0.7	1.3	125	77	39	71
13	M-138	F	p.Ala367Val	11.6	5	24.1	219	291	11	1	1.6	138	65	43	82
14	M-134	M	p.Pro379Ser	26	6.8	24	268	310	11.4	–	–	270	150	31	209
15	M-135	F	p.Pro379Ser	23	3	26.3	250	310	11.2	2.16	–	145	95	41	85
16	M-036	M	p.Pro379Ser	24	10	27.6	305	521	15.4	0.2	0.3	187	439	37	40
17	M-136	F	p.Pro379Ser	14	–	21.2	289	431	12.7	0.56	1.31	145	95	41	85
18	M-139	F	p.Asp602Asn	14	5	20	159	280	9	2	2.6	195	110	40	70
19	M-137	M	p.Leu611Pro	28.8	18.2	31.6	108	147	6	1.1	3	154	95	30	105
20	M-040	M	p.Glu619Lys	32	27	26.3	134	191	9.5	0.7	1.4	117	160	25	60

Among the 14 variants, four variants (p.Lys120Asn,p.Gln125His,p.Ala367Val,p.Asp602Asn) were novel and not reported in the literature, three variants were previously reported by us (3, 24), and the remaining seven variants were reported in other studies (20, 25–29). Of the 14 variants included in this study, six variants reside in DNA binding domain (91-281 a.a), specifically four variants were mapped to POU_S_ domain (91-181 a.a), one variant was mapped to POU_H_ domain (203-279 a.a) and one variant reside in the interface between the POUs and POU_H_ domains of *HNF1A* protein. The other, eight variants were mapped to the transactivation domain (282- 631 a.a) of *HNF1A* protein (**Supplementary Figure 2**).

### 3.2 Functional evaluation

#### 3.2.1 Altered transcriptional activity of *HNF1A* variants

**Figure 1:**
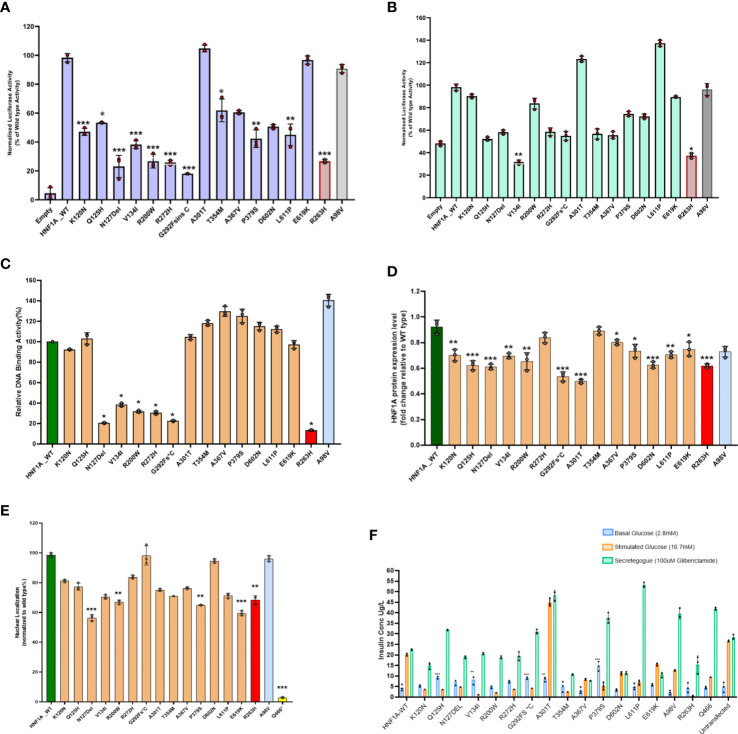
Summary of the data obtained from functional studies. **(A)** Transcriptional activity of the *HNF1A* protein variants in HeLa cells; **(B)** Transcriptional activity of the *HNF1A* protein variants in Ins1 cells using HNF4A P2 promoter; **(C)** Assessment of the DNA binding ability of the *HNF1A* protein variants; **(D)** Protein expression levels of the *HNF1A* protein variants in HeLa cells; **(E)** Nuclear localization of the *HNF1A* protein variants in HeLa cells; **(F)** Variant effect on Glucose Stimulated Insulin Secretion. Red bar indicates MODY 3 control variant; Grey bar indicates type 2 diabetes risk variant; Yellow bar indicates variant with poor nuclear translocation effect in *HNF1A* gene. Each bar represents the mean of three independent experiments (n=3) ± SD. P-values were obtained by un-paired student t-test. *** indicates p value <0.001; ** indicates p value <0.01; * indicates p value <0.05.

In HeLa cells compared to the WT *HNF1A* activity (set as 100%), the measured levels of transcriptional activity (TA) for five (p.Asn127*,p.Val134Ile,p.Arg200Trp and p.Gly292Fs*25)of the 14 variants were significantly lower (<40%) (**Figure 1A**, **Table 2**). Three variants (p.Lys120Asn,p.Pro379Ser, and p.Leu611Pro) had TA activity <50%, while two variants (p.Gln125His and p.Thr354Met) had TA activity of 53 and 62% respectively and reduction observed in all these variants were significant. Two variants p.Ala367Val (61%) and p.Asp602Asn (51%) showed a mildly reduced TA. Two other variants (p.Ala301Thr and p.Glu619Lys) demonstrated TA levels comparable to WT *HNF1A* levels (**Figure 1A**, **Table 2**). TA was consistently higher for all these variants when using *HNF4A-P2* promoter in INS-1 cells (activity range 32%–137%) (**Figure 1B**, **Table 2**) versus rat albumin promoter in HeLa cells. This is most likely due to interference of endogenous *HNF1A* in INS-1 cells (2- to 4-fold higher basal promoter activity).

**Table 2:** Summary of the functional studies of the *HNF1A* variants identified in Indian MODY subjects.

	S.No	Amino acid change at protein level	Nucleotide change at c.DNA level	Functional Study								Structure Prediction		
				Transactivation Assay (% WT)		DNA Binding Activity (% WT)	Protein Expression (% WT)	NuclearLocalisation (% WT)	GSIS (Insulin Levels)			Sequence Based Prediction	Structure Based prediction	Molecular Dynamics
				HeLa	Ins 1				Basal	Stimulated	On adding 100µM GBC			
**DNA Binding Domain**	1	p.K120N	c.360G>C	47	90	92	76	81	5	4	15	Destabilization effect	Higher Destabilization effect	Defect
	2	p.Q125H	c.375G>C	53	52	103	67	77	9	4	32	Destabilization effect	Least Destabilization effect	–
	3	p.N127del	c.377_379delACA	23	58	21	66	57	7	5	19	–	–	–
	4	p.V134I	c.400G>A	38	32	38	75	71	8	1	21	No defect	Least Destabilization effect	–
	5	p.R200W	c.598C>T	27	84	32	71	67	5	2	19	Destabilization effect	Higher Destabilization effect	No defect
	6	p.R272H	c.815G>A	26	59	31	91	84	7	4	19	Destabilization effect	Higher Destabilization effect	Defect
**Transactivation Domain**	7	p.G292fs*25	c.872-873dupC	18	55	23	58	98	9	4	31	–	–	–
	8	p.A301T	c.901G>A	105	123	105	54	75	8	45	48	–	–	–
	9	p.T354M	c.1061C>T	62	57	118	97	71	5	2	11	–	–	–
	10	p.A367V	c.1100C>T	61	56	130	87	76	3	8	8	–	–	–
	11	p.P379S	c.1135C>T	42	75	125	80	65	15	5	37	–	–	–
	12	p.D602N	c.1804G>A	51	72	115	68	95	3	11	11	–	–	–
	13	p.L611P	c.1832T>C	45	137	112	76	71	5	7	25	–	–	–
	14	p.E619K	c.1855G>A	97	90	97	81	60	6	16	11	–	–	–
	15	p.Arg263His	c.788G>A	27	37	13	67	69	4	1	15	–	–	–
	16	p.Ala98Val	c.293C>T	91	96	141	76	96	2	13	26	–	–	–
	17	p. Gln466*	c.1396 C>T	_	_	_	_	7	_	_	_	–	–	–

Shaded in grey are used as control for the functional assay.

#### 3.2.2 Effect of variants on DNA- binding activity of *HNF1A* to target DNA sequence

Three variants (p.Asn127*, p.Arg200Trp and p.Arg272His) localized in the DBD and one variant (p.Gly292Fs*25) in TAD demonstrated severely reduced (<40%) activity. All other variants showed normal binding activity comparable to WT (**Figure 1C**, **Table 2**).

#### 3.2.3 Effect of variants on *HNF1A* protein expression

Two variants (p.Gly292Fs*25 and p.Ala301Thr) showed significantly reduced protein expression level (<60%); while four variants (p.Gln125His,p.Asn127*,p.Arg200Trp and p.Asp602Asn), demonstrated reduced expression level (61-75%) and were also significant (**Figure 1D**, **Table 2**).

#### 3.2.3 Effect of variants on nuclear localization of *HNF1A* protein

All the 14 *HNF1A* variants were assessed for their ability to translocate to the nucleus of the cell in order to regulate their target gene expression. Only four variants showed reduced (~57-67%) nuclear translocation as assessed by indirect immunocytochemistry (**Figure 1E**, **Table 2**). Other variants showed normal nuclear translocation.

#### 3.2.4 Effect of variants on insulin secretion

All 14 variants were also assessed for insulin secretion using GSIS. Under basal conditions (2.8mM glucose), these variants produced insulin in the range of 3-15µg/L of insulin and under stimulated conditions using 16.7mM glucose they produced 1-45µg/L of insulin. When they were treated with 100µM glibenclamide (GBC), the stimulated insulin secretion was enhanced ranging from 8-48µg/L in all the 14 variants tested (**Figure 1F**, **Table 2**).

### 3.3 Structural evaluation

Structural analysis was performed for variants found in DNA binding domain. These variants were mapped onto the crystal structure of *HNF1A* protein (PDB ID: 1IC8). Thereby, all the missense variants, namely p.Lys120Asn, p.Gln125His, p.Val134Ile, p.Arg200Trp, and p.Arg272His, were subjected to the following predictions such as sequence and structural-based stability prediction followed by molecular dynamics (MD).

**Figure 2:**
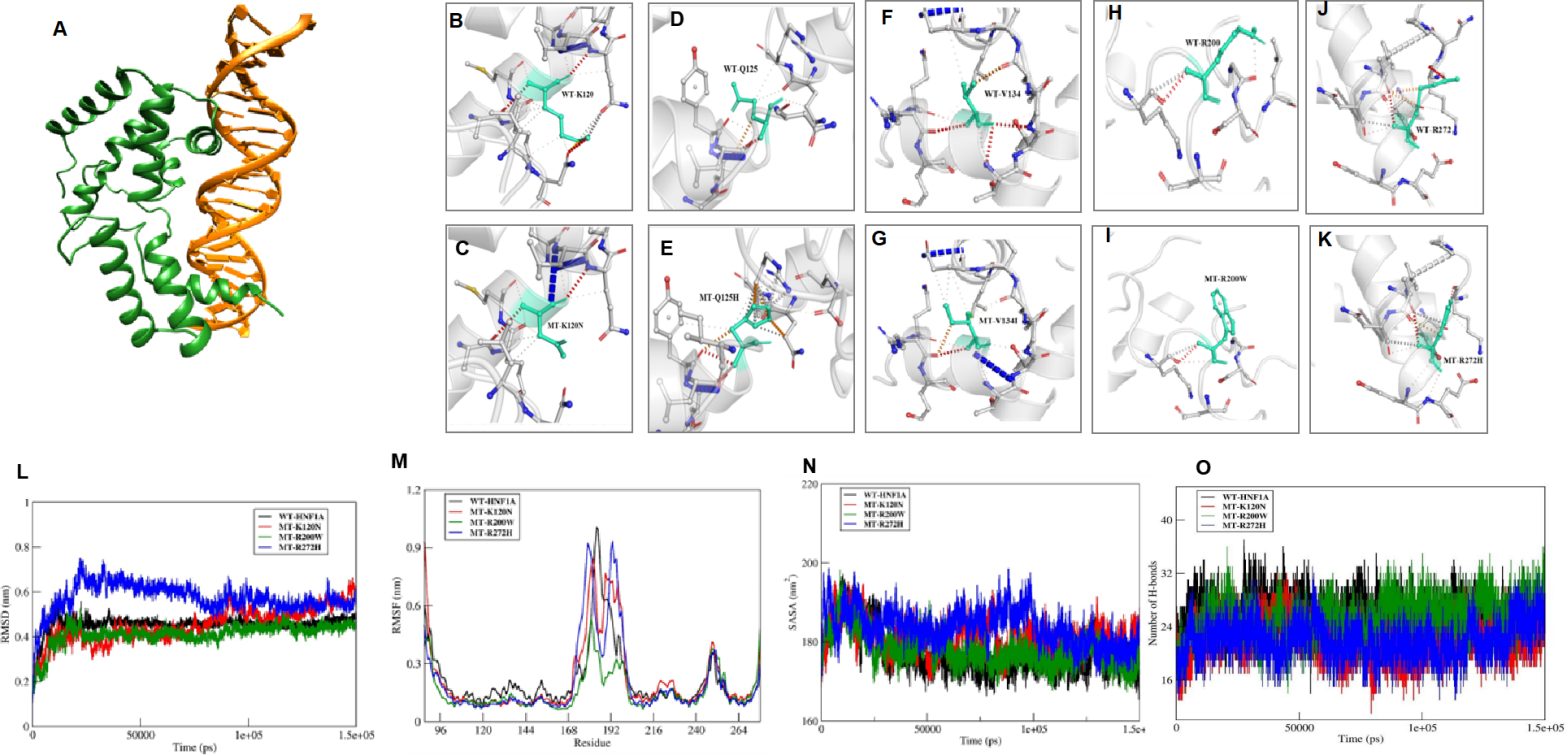
*In Sillico* structural prediction of wild type and variant *HNF1A* protein. **(A) **Remodelled and Refined Wild-type (WT) *HNF1A*-DNA complex; **B-K**)Prediction of Interatomic Interactions of the Wild and mutant forms of *HNF1A* variants, where the Wild-type and mutant residues are coloured in light-green and are also represented as sticks alongside the surrounding residues which are involved in any type of interactions; **(L-O)** Molecular dynamics simulation analysis of the wild and MT forms of *HNF1A* complexes **(L)** RMSD plot **(M)** RMSF plot **(N)** Solvent accessible surface area plot; **(O)** Number of inter hydrogen bonds maintained throughout the MD production run within *HNF1A* and DNA.

Sequence-based stability study revealed that the *HNF1A* structure is destabilized by the variants p.Lys120Asn, p.Gln125His, p.Arg200Trp, and p.Arg272His, but not by the variant p.Val134Ile. The crystal structure of *HNF1A* in association with DNA (PDB ID-1IC8), was further modified with missing residues and refined using Modeller10v for the structure-based stability prediction (**Figure 2A**). According to structure-based prediction, the *HNF1A* variants **p.Lys120Asn, p.Arg200Trp, and p.Arg272His** were shown to have a larger destabilizing impact and more molecular flexibility than the other variants. Among these variants, the p.Arg200Trp variant has a higher destabilizing impact. Variants p.Gln125His and p.Val134Ile had the least destabilizing impact (**Figures 2B–K**). Since the three variants p.Lys120Asn, p.Arg200Trp and p.Arg272His, showed higher destabilizing effects they were chosen for the MD study.

#### 3.3.1 Molecular dynamics stability analysis of the wild and mutant complexes

The WT-*HNF1A* template was used to simulate the structures of the mutants p.Lys120Asn, p.Arg200Trp, and p.Arg272His. The revised WT and MT *HNF1A* were then submitted to MD simulation investigations using Gromacs2020. When the complexes’ MD trajectories were compared to the WT, the variant p.Arg272His showed higher divergence than the variants p.Lys120Asn and p.Arg200Trp in the initial period of simulation. However, variant p.Lys120Asn showed more deviations than p.Arg272His during the last 20 ns of the root mean square deviation (RMSD) plot, a numerical measurement representing the difference between WT and variant protein structures (**Figure 2L**). The root mean square fluctuation (RMSF) plot, is a calculation of individual residue flexibility, or how much a particular residue moves (fluctuates) during a simulation (**Figure 2M**), and this showed that residues that interact with DNA were found to have larger deviations in all of the complexes; in particular, residues 179 and 180 of the p.Arg272His variant showed higher deviations of 0.9 nm and 192-193 of the p.Arg272His variant showed higher fluctuations of about 1 nm among the complexes. When compared to WT, the variants p.Lys120Asn and p.Arg272His lost their contact with DNA at the residue level, and their total interactions with DNA also decreased (**Figures 2N, O**). However, the variant p.Arg200Trp had an increased frequency of interactions with DNA and a greater accessible surface area of all buried solvents (**Figures 2N, O**). Particularly, the variant residue Trp200 interacts with the minor groove of DNA. From these results, it was revealed that variants p.Lys120Asn and p.Arg272His had lost their interaction with DNA resulting in structural defects.

### 3.4 Reinterpretation of *HNF1A* variants based on molecular characterization

Pathogenic *HNF1A* variants causing *HNF1A*-MODY are often characterized by significantly decreased TA, poor DNA binding, impaired nuclear targeting, and/or lower protein expression levels in the range of ~20-35% when compared to WT (100%) (19, 21, 30–33). In this study, the cut-off considerations were set at a slightly different level compared to the previous study by Althari et al. (31). Being a more distilled cohort of clinically proven MODY patients, the cut-off of TA<40% was used for pathogenic variants, and TA activity between 40-60% was used for likely pathogenic variants. In addition to this, DNA binding activity, GSIS, and clinical course were considered for ascribing pathogenic and likely pathogenic variants. Therefore, over and above the ACMG/AMP guidelines, the functional and clinical work such as the response to SU have been considered together to re-interpret the variants.

**Figure 3:**
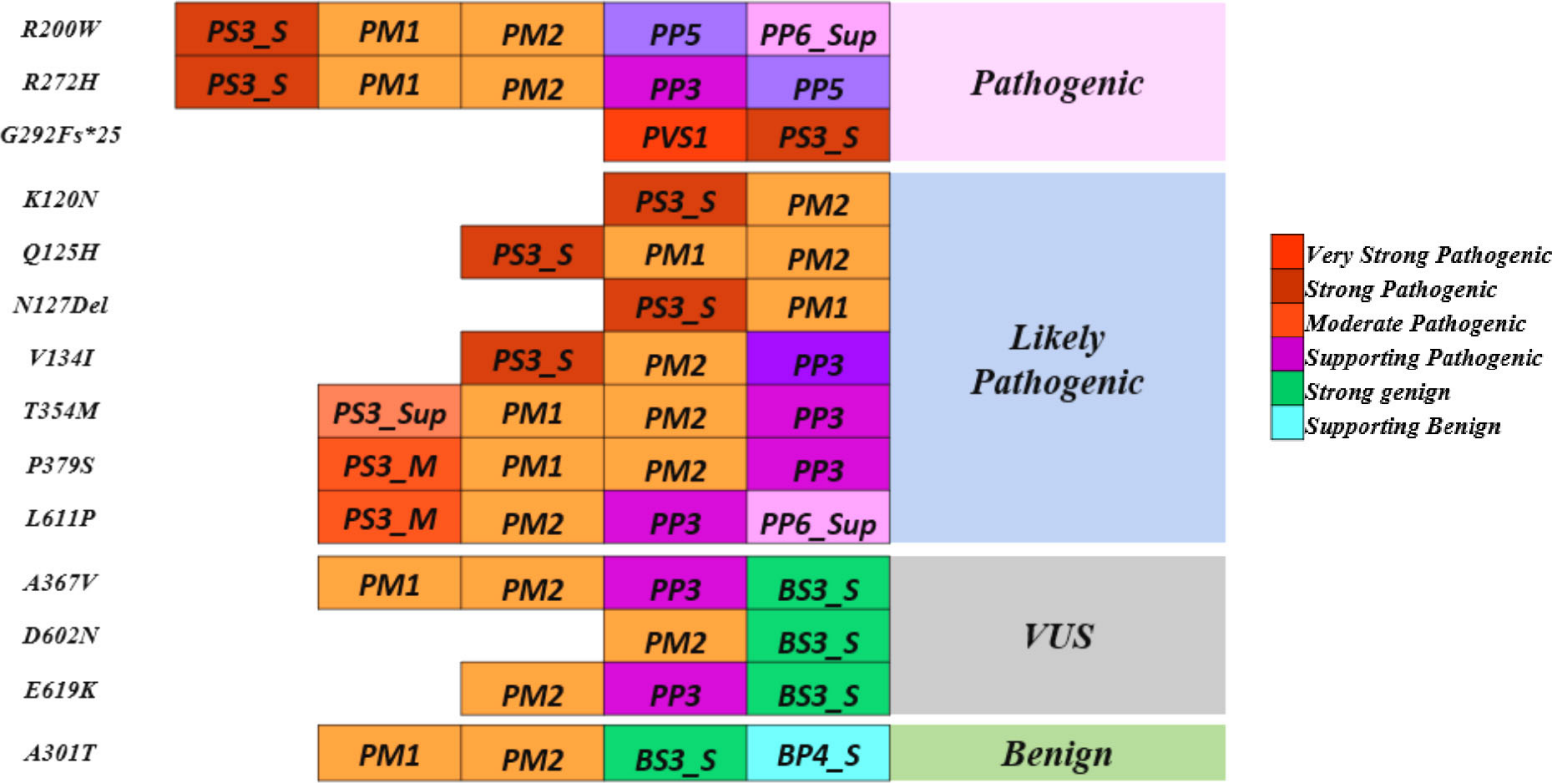
Reinterpretation of *HNF1A* variants using molecular characterization.

Variants p.Gly292Fs*25 and p.Asn127* were interpreted as pathogenic variants since they have low TA activity along with the reduced DNA binding activity and defect in insulin secretion.p.Arg272His was reinterpreted as a pathogenic variant from their initial interpretation. Seven variants (p.Lys120Asn, p.Gln125His, p.Val134Ile, p.Arg200Trp, p.Thr354Met, p.Pro379Ser, and p.Leu611Pro) were reclassified as likely pathogenic variants from VUS. Three variants (p.Ala367Val, p.Asp602Asn, and p.Glu619Lys) remained VUS after reinterpretation whereas variant p.Ala301Thr was reinterpreted as benign from VUS (**Figure 3**, **Table 3**).

**Table 3:** Summary of re-interpretation of *HNF1A* gene variants and their clinical actionability, identified in Indian MODY patients based on molecular characterization.

	S.No	Amino acid change at protein level	Nucleotide change at c.DNAlevel	Variant Interpretation_ ACMG guidelines 2015		Functional Study								Structure Prediction			Reinterpation Based on functional evidence		Clinical Actionability
						Transactivation Assay (% WT)		DNABinding Activity (% WT)	Protein Expression (% WT)	NuclearLocalisation (% WT)	GSIS (Insulin Levels)			Sequence Based Prediction	Structure Based prediction	Molecular Dynamics			
				Evidence	Classification	HeLa	Ins 1				Basal	Stimulated	On adding 100µM GBC				Evidence	Classification	
**DNA Binding Domain**	1	p.K120N	c.360G>C	PM1,PM2	VUS	47	90	92	76	81	5	4	15	Destabilization effect	Higher Destabilization effect	Defect	PS3_Moderate, PP3_Strong	LP	Actionable
	2	p.Q125H	c.375G>C	PM1,PM2	VUS	53	52	103	67	77	9	4	32	Destabilization effect	Least Destabilization effect	–	PS3_Moderate,PP3 and PP6	LP	Actionable
	3	p.N127del	c.377_379delACA	PM1,PM2	VUS	23	58	21	66	57	7	5	19	–	–	–	PS3_Strong	P	Actionable
	4	p.V134I	c.400G>A	PM1,PM2,PP3	VUS	38	32	38	75	71	8	1	21	No defect	Least Destabilization effect	–	PS3_Strong	LP	Actionable
	5	p.R200W	c.598C>T	PM1,PM2,PP5	VUS	27	84	32	71	67	5	2	19	Destabilization effect	Higher Destabilization effect	No defect	PS3_Strong	P	Actionable
	6	p.R272H	c.815G>A	PM1,PM2,PP3, PP5	LP	26	59	31	91	84	7	4	19	Destabilization effect	Higher Destabilization effect	Defect	PS3_Strong	P	Actionable
**Transactivation Domain**	7	p.G292fs*25	c.872-873dupC	PVS1	LP	18	55	23	58	98	9	4	31	–	–	–	PS3_Strong	P	Actionable
	8	p.A301T	c.901G>A	PM1,PM2	VUS	105	123	105	54	75	8	45	48	–	–	–	BS3_Strong, BP4_Strong	B	_
	9	p.T354M	c.1061C>T	PM1,PM2,PP3	VUS	62	57	118	97	71	5	2	11	–	–	–	PS3_Supporting	LP	Actionable
	10	p.A367V	c.1100C>T	PM1,PM2	VUS	61	56	130	87	76	3	8	8	–	–	–	BS3_Strong, BP4_Strong	VUS	Unresolved
	11	p.P379S	c.1135C>T	PM1,PM2,PM5,PP3	LP	42	75	125	80	65	15	5	37	–	–	–	PS3_Moderate	LP	Actionable
	12	p.D602N	c.1804G>A	PM1,PM2	VUS	51	72	115	68	95	3	11	11	–	–	–	BS3_Strong	VUS	Unresolved
	13	p.L611P	c.1832T>C	PM1,PM2,PP3	VUS	45	137	112	76	71	5	7	25	–	–	–	PS3_Moderate	LP	Actionable
	14	p.E619K	c.1855G>A	PM1,PM2,PP3	VUS	97	90	97	81	60	6	16	11	–	–	–	BS3_Strong	VUS	Unresolved

P, Pathogenic; LP, Likely Pathogenic; B, Benign; VUS, Variant of Uncertain significance.

### 3.5 Clinical follow-up of the patients with *HNF1A* variants

Variants designated as pathogenic/likely pathogenic based on functional assessment were investigated for clinical actionability by collecting the follow-up details of the patients over a period of time. The patient (M-026) with variant p.Lys120Asn has been switched from insulin to two doses of SU (glimepiride) along with metformin per day. The patient M-027 with the mutation p.Gln125His (likely pathogenic variant) developed diabetes at the age of 25.7 years and had diabetes for 7 years. Before genetic testing, the patient was treated with insulin and oral hypoglycemic agents (OHA). As a result of genetic studies, the patient was transferred from insulin to two doses of gliclazide per day. His HbA1C levels dropped from 9.6% to 6.4% after his therapy was changed.

**Figure 4:**
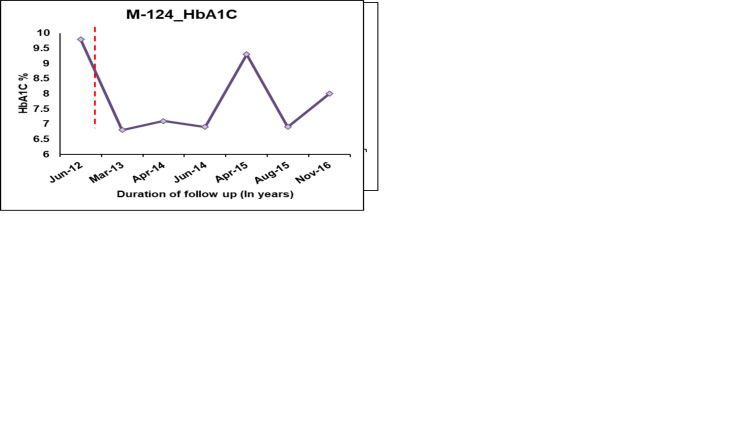
HbA1C Trajectories of few *HNF1A* MODY patients after change in treatment based on re-interpretation of the variants. Dotted lines indicate change of treatment.

Patient M-028, who carries the pathogenic variant p.Asn127*, is diagnosed with diabetes at the age of 14.9 years, with a duration of 15.6 years (**Figure 4**). The patient was on OHA for around two years before being started on insulin. She is currently on insulin and SU therapy since her β cell reserve was low (CPF-0.6 and CPS-0.9) and she started to develop microvascular and macrovascular complications. Patient M-124 harboring the variant p.Val134Ile (Likely pathogenic variant) was diagnosed with diabetes at the age of 26.7 years with diabetes duration of 4 years. Based on functional evidence, patient M-124 with variant p.Val134Ile was transitioned from insulin to a single dose of glipizide per day.

Patient M-126 with the pathogenic variant p.Arg200Trp was switched from insulin to SU. It was advised to continue with SU for patient M-125 who had the same variant. Statins were given for patient M-125 in order to maintain a normal lipid profile. Previous studies have shown two other amino acid changes at the same codon such as p.Arg200Gly and p.Arg200Gln in multiple SU-sensitive *HNF1A*-MODY families (34, 35). The functional effects of these two variants, p.Arg200Gly and p.Arg200Gln, were however not mentioned. All of the patients, including the one from this study, who have the variation in this codon respond to SU. This suggests that the variation is pathogenic and clinically actionable. Patients with pathogenic variant (p.Arg200Trp, p.Arg272His and p.Gly292Fs*25) and likely pathogenic variant (p.Thr354Met and p.Leu611Pro) were also shifted from insulin to SU therapy.

## 4 Discussion

The comprehension of disease mechanisms is improved by well-established functional investigations on variants, which also offer proof for the pathogenicity of the variants. Studies have demonstrated that functional studies help to clarify the interpretation of *HNF1A*-MODY variants, particularly in the absence of familial segregation or phenotypic data (32).

In this study, we have performed molecular characterization of 14 *HNF1A* variants identified in 20 unrelated individuals from 20 non-consanguineous families among Indian MODY subjects, where the majority of variants have not been reported. Normal transactivation activity of *HNF1A* protein, which depends on the capacity to bind target promoters (DNA) and on an adequate quantity of cellular (nuclear) protein, is necessary for normal *HNF1A* transcription factor function.

Because not all functional tests represent the underlying process and not all variants have the same effects on function (36), we aimed at improving the understanding and interpretation of these findings. Therefore, multiple assays were employed to fully examine the effects of a variant in order to come to a conclusion. These variants were examined utilizing *in vitro* functional pipelines, such as luciferase assays for transactivation, which measure the transcriptional activity of *HNF1A* variants, as well as assays of DNA binding activity, protein expression, and subcellular localization to determine the impact of the variants on the protein function. Additionally, a GSIS assay to examine the impact of these variants on insulin secretion was performed. A distinctive feature of this work is the *in silico* structural analyses to determine if it might identify the variants with functional defects. Since the crystal structure of *HNF1A* is available only for the DNA binding domain, structural investigations were carried out for the missense variants identified only in that region.

A multi-pronged approach using the ACMG guidelines, the functional and structural analyses have been considered together to re-classify these variants. In this work, we focused on the scoring systems and the criteria for re-interpreting the variants. PS3 was assigned when data from well-established *in vitro* functional studies supported a detrimental effect on the gene or gene product; PP3 was assigned when multiple lines of computational evidence and structural prediction supported a detrimental effect on the gene or gene product (conservation, evolutionary, etc.); and BS3 was assigned when well-established *in vitro* functional studies showed no detrimental effect on protein function. In addition, multiple levels of strength, such as strong, moderate, and supporting levels based on functional and structural data were applied to the scoring approaches employed in this study. Of the 14 variants considered in this study, 1 variant p.Arg272His was interpreted as likely pathogenic, and 11 variants were interpreted as VUS initially based on the ACMG/AMP guidelines. (**Figure 3**, **Table 3**).

According to previous studies on the effects of pathogenic *HNF1A*-MODY variants, pathogenic and MODY causal variants impair *HNF1A* activity, DNA binding, and localization (40% compared to WT *HNF1A*) (21, 32), whereas type 2 diabetes risk variants have an impact on *HNF1A* function ranging from 40% -60% compared to WT (30, 31, 33).

Based on the aforementioned cut-offs, many degrees of strength were assigned to each scoring criterion. PS3_Strong scoring criteria were assigned to variants that showed <40% activity than WT activity in at least two functional assays; PS3_Moderate was assigned to variants that showed activity between 40 and 60%; and PS3_Supporting was assigned to variants that showed activity less than 65%. PP3_Strong criterion was assigned when the variant showed defects in all the *in silico* structural prediction analysis. The variant meeting the BS3_Strong criterion had no negative effect on protein function in any of the functional experiments.

The *p.Arg272His* previously interpreted as likely pathogenic was re-interpreted as *pathogenic* based on the evidence *PS3_Strong, PM1, PM2, PP5, and PP3_Strong*. One variant *p.Arg200Trp* interpreted as VUS was re-interpreted as *pathogenic* based on the evidence *PS3_Strong, PM1, PM2, PP3_Supporting, and PP5*. Variant p.Gly292Fs*25 was interpreted as *pathogenic* based on the evidence *PVS1 and PS3_Strong* and variant p.Asn127* was interpreted as *likely pathogenic* based on the evidence *PS3_Strong, PM1.* Variants *p.Lys120Asn* and *p.Gln125His* interpreted as VUS was re-interpreted into *likely pathogenic* based on the evidence *PS3_Moderate, PM2, PP3_Strong, and PS3_Moderate, PM2, PP3_Supporting, PP6* respectively. Variant *p.Val134Ile* was re-interpreted into *likely pathogenic* based on evidence *PS3_Strong* and *PM2*. Variant *p.Thr354Met* was re-interpreted as likely pathogenic based on *PS3_Supporting, PM1, PM2, and PP3*. Variant *p.Pro379Ser* was re-interpreted as *likely pathogenic* based on the evidence *PS3_Moderate, PM1, PM2, and PP3*. Variant *p.Leu611Pro* was re-interpreted as *likely pathogenic* based on the evidence *PS3_Moderate, PM2, PP3, and PP6_Supporting*. Variant *p.Ala367Val* remains *VUS* based on the evidence *PM1, PM2, PP3, and BS3_Strong*. Variants *p.Asp602Asn* and *p.Glu619Lys* remain *VUS* based on the evidence *PM2, BS3_Strong and PM2, PP3, and BS3_Strong* respectively. Variant *p.Ala301Thr* was re-interpreted as *benign* based on the evidence *PM1, PM2, BS3_Strong, and BP4_Strong* (**Table 3**). It is crucial to remember that functional evidence does not always associate a variant to disease outcome; in order to determine clinical actionability, the functional data must be assessed in combination with clinical data (30). It is important to be aware of the fact that both functional and longitudinal clinical follow up are important to establish the clinical actionability of the variants.

Clinical actionability is generally defined as clinically prescribed interventions that are effective for preventing or delaying clinical disease, lowering clinical burden, or improving clinical outcomes in an adult who has not previously received a diagnosis and are specific to the genetic disorder under consideration (37). Based on our results, 4 out of 14 (28.6%) variants were interpreted as pathogenic, 6 variants (42.8%) as likely pathogenic, 3 variants (21.4%) as variants of uncertain significance, and 1 variant (7.14%) as a benign variant. Patients with the ten P/LP variants were able to successfully switch from insulin to SU and sustain good glycemic control, thus making these variants clinically actionable (**Table 3**).

We performed 3D structural analysis to check whether *in-silico* analysis corroborated with functional investigations in identifying the pathogenic variants and also to have a structural understanding of the variant *HNF1A* proteins. Our *in-silico* analysis showed that variants p.Gln125His, p.Val134Ile have lesser structural defects while variants p.Lys120Asn and p.Arg272His have severe structural defects, and the variant p.Arg200Trp has moderate structural defects. In the case of the p.Val134Ile variant, we found differences between the functional and structural data. Although *in-silico* structural analysis showed that it has a lesser destabilizing effect despite being predicted to be a highly conserved structural residue, our functional data showed that variant p.Val134Ile has a defect in DNA binding thus down-regulating the target genes resulting in reduced insulin secretion (**Table 2**). Moreover, the patient follow-up also showed that the patient (M-124) responded well to treatment change to SU, making this variant a clinically actionable one (**Figure 4**).

Our study has a few limitations. Since we could not obtain family samples for many patients, we were unable to conduct family co-segregation studies. In some patients, we did not have adequate clinical data.

In summary, this paper exemplifies the importance of performing molecular characterization after genetic testing, since the understanding of the functional basis of genotypes helps in understanding the phenotype which could lead to changes in clinical treatment for monogenic disorders like MODY. Our findings are the first to show the need of using additive scores during molecular characterization for accurate pathogenicity evaluations of *HNF1A* variants in precision medicine. Furthermore, it is also one of the first to introduce structural understanding to functional implications. The study has led to the delineation of the VUS into pathogenic and disease-causing MODY variants, from non-pathogenic variants. Patients with most pathogenic *HNF1A* variants benefit from OHA treatment; hence, this would assist clinicians in determining the best course of action for patients. While the combination of functional and structural-based approaches may lead to increased certainty in variant–phenotype correlation in a research setting, a functional understanding of the variants helps in precision diagnosis and treatment in a monogenic disorder such as MODY.

## Data availability statement

The datasets presented in this study can be found in online repositories. The names of the repository/repositories and accession number(s) can be found in the article/**Supplementary Material**.

## Ethics statement

The studies involving human participants were reviewed and approved by Institutional ethics committee, MDRF. Written informed consent to participate in this study was provided by the participants’ legal guardian/next of kin.

## Author contributions

VR and BK designed and implemented the functional study. BK analyzed the data and wrote the manuscript. SR designed and performed the structural analysis. UV and NH analyzed the structural data. SG performed segregation analysis. VM collected the clinical data and analyzed the manuscript. VR analyzed all data and corrected the manuscript. All authors contributed to the article and approved the submitted version.
