# Supplementary material for: Molecular characterization and re-interpretation of HNF1A variants identified in Indian MODY subjects towards precision medicine
Source: Front Endocrinol (Lausanne). 2023 Jun 16;14:1177268. doi: 10.3389/fendo.2023.1177268 (PMC10313120; doi:10.3389/fendo.2023.1177268)
Supplement: Supplementary file 1 [file DataSheet_1.pdf]

Supplementary Material

MOLECULAR CHARACTERIZATION AND RE-INTERPRETATION OF *HNF1A* VARIANTS IDENTIFIED IN INDIAN MODY SUBJECTS TOWARDS PRECISION MEDICINE

Babu Kavitha[1], SampathKumar Ranganathan[2], Sundaramoorthy Gopi[1], Umashankar Vetrivel [3,4], Nagarajan Hemavathy [3], Viswanathan Mohan[5], ***Venkatesan Radha[1]****

Affiliations:

1. Department of Molecular Genetics, Madras Diabetes Research Foundation, ICMR Centre for Advanced Research on Diabetes, University of Madras, Chennai, India.
2. Centre for Bioinformatics, School of Life Sciences, Pondicherry University, Puducherry, India
3. Department of Bioinformatics Vision Research Foundation, No.18/41, College Road, Nungambakkam, Chennai, Tamil Nadu, India
4. ICMR-National Institute of Traditional Medicine, Nehru Nagar, Belagavi, Karnataka-590 010, India.
5. Department of Diabetology, Madras Diabetes Research Foundation, Chennai & Dr. Mohan's Diabetes Specialties Centre, IDF Centre of Education, Chennai, India

***Corresponding Author and Name and address of author to whom reprint requests should be addressed:**

Venkatesan Radha, Ph.D.

Department of Molecular Genetics, Madras Diabetes Research Foundation,

ICMR Centre for Advanced Research on Diabetes, Affiliated to University of Madras, Chennai, India.

#4, Conran Smith Road, Gopalapuram, Chennai 600 086, India.

Tel.: +91 44 47405900, 91 44 43968888; Fax: +91 44 28350935;

E-mail: radharv@yahoo.co.in , drradha@mdrf.in

1. Supplementary Data:

Detailed Functional Characterization methods:

1.1 Functional Analysis:

1.1.1 Plasmids:

Human *HNF1A* cDNA (NCBI Entrez Gene BC104910.1) (NM_000545.5) in pcDNA 3.1 His/C vector (Invitrogen Inc, Carlsbad, CA, USA), was used as a template for

constructing individual *HNF1A* variants using the QuikChange Lightning Site-directed Mutagenesis Kit (Agilent Technologies, Santa Clara, CA), and all constructs were verified by Sanger sequencing.

Two reporter constructs were used for transactivation experiments: (1) pGL3-RA, which contains the promoter of the rat albumin gene (nucleotide -170 to +5) next to the Firefly luciferase gene in vector pGL3-Basic (Promega), and (2) pGL3-HNF4AP2, which contains the human *HNF4A* (MIM: 600281) P2 promoter (nucleotide -418 to +13). pRL-SV40 reporter vector encoding the Renilla luciferase gene was used as an internal control (Promega).

1.1.2 Cell culture and Transfection

The transactivation experiment was performed on HeLa and INS-1 832/13 cell lines. HeLa cells were grown in Dulbecco's Modified Eagle Medium (Gibco Life Technologies, NY, USA) supplemented with penicillin–streptomycin solution (100 U/ml) and 10% heat-inactivated Fetal Bovine Serum (Gibco). INS-1 832/13 cells were cultured in RPMI-1640 medium (Sigma: R0883) with 2 mM L Glutamine (TMS-002-C), 1 mM sodium pyruvate (TMS-005-B), 10 mM HEPES (TMS-003-C), 0.05 mM mercaptoethanol (ES-007-E), and 10% FBS (ES-009-B). Twice a week, cells were divided in a 1:10 ratio.

The day before transfection, cells were divided into 12-well culture plates to reach 80–85 % confluency at the time of transfection.3 µl of Fugene HD (Roche, Indianapolis, IN, USA) transfection reagent and a total of 1 µg of plasmid DNA (0.25 µg of WT or mutant HNF1A plasmid, 0.25 µg of pGL3-RA reporter gene construct, 0.4875 µg of empty pcDNA3.1 His/C vector and 0.0125 µg of control vector pRL SV40) served as optimal conditions for the transfection.

1.1.3 Transactivation Assay:

The transcriptional activity (TA) of *HNF1A* variants were assessed in HeLa cell line representing cells negative for endogenous *HNF1A* expression and in INS-1 (rat insulinoma

pancreatic cell line as β-cell model). Transactivation assay was performed as described in [14] Balamurugan et al., 2016. In brief, 24 h post-transfection, cells were lysed with 1X Passive Cell Lysis Buffer (Promega) and the cell lysate was used to measure the luciferase activity using dual luciferase reporter assay kit (Promega) in SpectraMax i3X platform (Molecular Device). Luciferase activity was normalized for transfection efficiency by the Renilla luciferase activity. All transactivation experiments included three parallels performed on three independent experimental days (nine readings in total).

1.1.4 DNA Binding Assay

A double-stranded biotinylated oligonucleotide of *HNF1A* binding site in the rat albumin promoter (5'-TGTGGTTAATGATCTACAGTTA-3') was used as a probe. Nuclear extracts were prepared by fractionation method and the total protein in each sample was quantified by BCA protein assay. Protein-DNA binding was analyzed by Episeeker DNA-protein binding assay kit (Abcam, ab117139) followed by manufacture's protocol. Briefly, 40 ng of probe and 50ug of nuclear extract were incubated on streptavidin-coated plate. The extract was then removed and washed with 1X washing buffer. Finally, the Xpress-tagged HNF1A was detected with anti-xpress (Invitrogen, USA) and HRP-conjugated anti-mouse-IgG (Santa Cruz Biotechnology) using TMB as substrate. Binding was assessed by reading the absorbance on amicro-plate reader at 450 nm.

1.1.5 Immunoblotting

The level of wild-type and individual HNF1A variant protein expressions in total HeLa cell lysates was determined. Total protein was quantified using BCA method (Thermo Scientific, US) and 10 - 30 μg of total protein per variant was denatured at 80°C for 20 minutes and then loaded onto a 4-20% Criterion TGX Stain-free Precast gel (Bio-Rad, Hercules, US) and electrophoresed. Then immunoblotted with antibodies for HNF1A (Cell Signaling) and beta-actin (Santa Cruz Biotechnology) and visualized using the ChemiDoc Imaging System (Bio-Rad). The blots were then scanned and densitometry of the protein

bands was analyzed using ImageJ Lab Software (Bio-Rad).

1.1.6 Sub cellular Localization

HeLa cells were platted at 20 to 35% of confluency into 12 well culture plate containing 18mm cover slips, pre coated with poly L-Lysine (Biokeystone, US). After 48hrs of post transfection, cells washed twice with 1xPBS and fixed with 4% paraformaldehyde for 20mins. Permeabilized with 0.1% Tween 20 and 0.1% Triton X 100 for 30mins. Then, cells were blocked with 5% blocking solution for 2 hours. The Immunofluorescence assay was performed using anti Xpress antibody (Invitrogen, USA) as a primary antibody, and anti-mouse IgG secondary antibody (Alexa flour 488, Thermo, USA). Nuclei stained by fluoroshield with DAPI (Sigma, USA). Nuclear –DAPI and HNF1A protein-stained cells were visualized by using a LSM 700 Flexible Confocal microscope with appropriate filters system (Carl Zeiss, US). The *HNF1A* variant c.1396 C>T (p.Q466*), was included as a positive control for impaired nuclear localization (cytosolic retention).

1.1.7 Insulin Secretion

The glucose-stimulated insulin secretion (GSIS) capacity of the INS1 β-cells were measured using insulin ELISA kit (Mercodia, Sweden). After appropriate treatment for 24 h, INS1 cells were washed with Kreb's Ringer Buffer (KRB) and then pre-incubated in the same buffer containing 2.5 mM glucose for 1 h. The pre-incubation buffer was removed and fresh KRB buffer with 2.5 mM (low) glucose or 16.5 mM (high) glucose, (100μM Glibenclamide) was added and incubated for 1 h. After incubation, supernatant was taken for insulin ELISA. ELISA was performed as per the manufacturer's instructions. Briefly, 10 μL of the calibrators, controls, and samples were added to the coated wells. 100 μL of enzyme conjugate solution was added to all the wells and incubated for 2 h on shaker at room temperature. After washing 6 times with wash solution, 200 μL of substrate was added to all the wells and incubated for 15 minutes at room temperature. Then 50 μL of stop solution was added and shaken for 5 sec. The optical density (OD) was measured at 450 nm using multimode plate reader (PerkinElmer,

Waltham, MA, USA). The concentration of insulin secreted under different experimental conditions was determined by plotting a standard graph with known concentrations of the calibrators used.

1.2 In silico analysis of the variants:

Among the 6 mutants of Hepatocyte nuclear factor 1-alpha (HNF1A) (Table 1), one mutation that occurs at Asn127 is a deletion mutation. Apart from the deletion mutation, the 2 mutants namely R200W (rs193922598), and R272H (rs137853238) are well-known mutants of HNF1A. Thereby, all the missense non-synonymous mutations namely, K120N, Q125H, V134I, R200W, and R272H were subjected to sequence-based pathogenicity, structural stability, and functional impact of these mutations subsequently. Prior to pathogenicity and stability analysis, HNF1A was subjected to conservation analysis using Consurf. Following this, all the selected mutants were subjected to pathogenicity analysis using PredictSNP. In addition, their effects on protein stability were calculated using three in silico prediction tools. I-Mutant 2.0, SDM, mCSM, and DUET. Finally, their impact on functionality was predicted using the Mutpred server. Based on the overall analysis of the mutants, only the ones that are pathogenic and have a higher impact on protein stability and functionality were modelled based on wild-type HNF1A (PDB ID-1IC8) which was remodelled and refined using Modeller10v. The refined Wild type (WT) HNF1A and mutants were subjected to molecular dynamics simulation studies using Gromacs2020 (10.1080/07391102.2021.1965030) Subsequently, PCA and FEL analysis was carried out to determine the near-native conformation, wherein the HNF1A-DNA interactions were analysed using DNAproDB.

2. Supplementary Figures and Tables

2.1: Supplementary Figures:

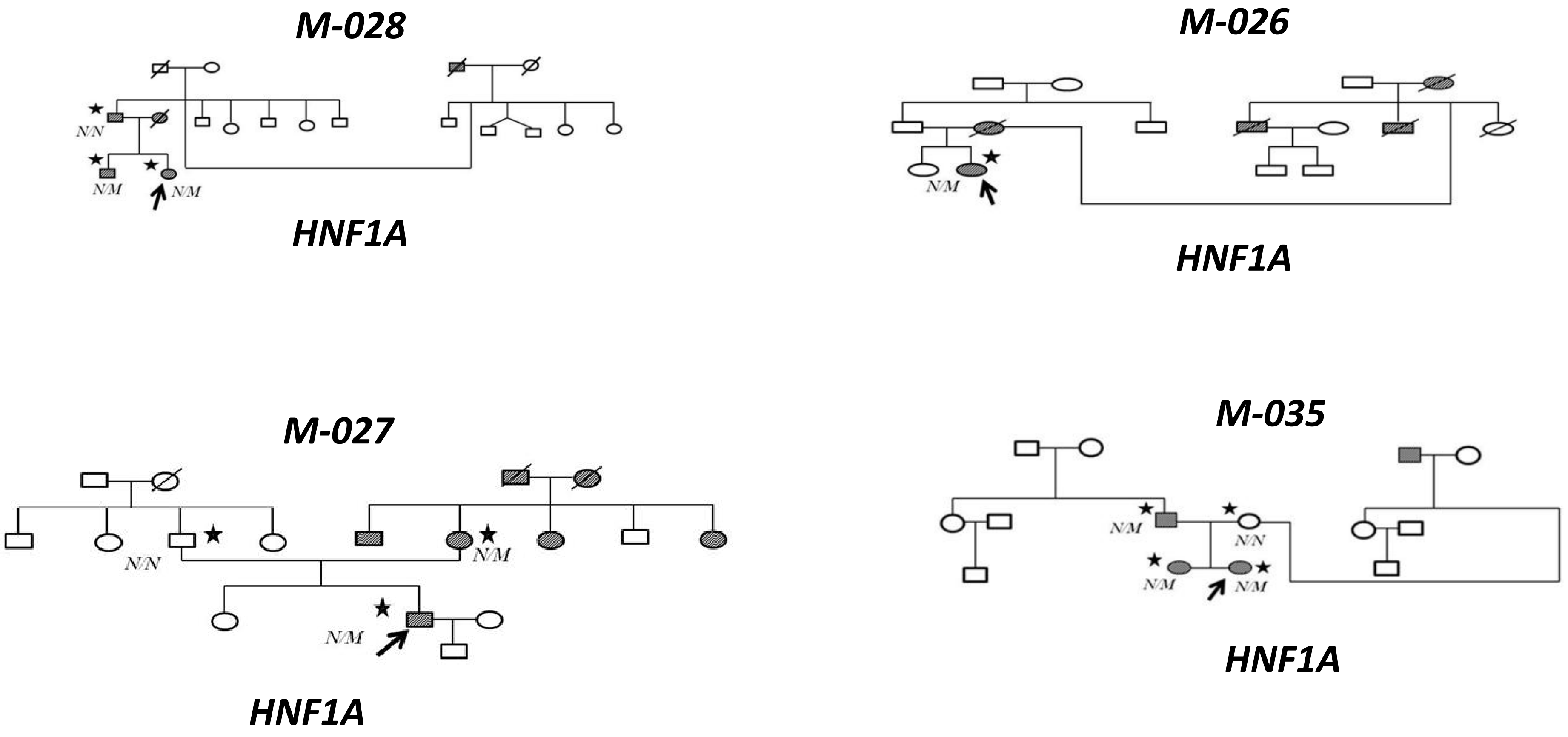

Supplementary Figure 1 : Pedigrees of MODY patients with variations in HNF1A gene. Pedigrees of the families showing diabetes status of each member, as well as genetic status: M/N (heterozygous), N/N (homozygous normal).

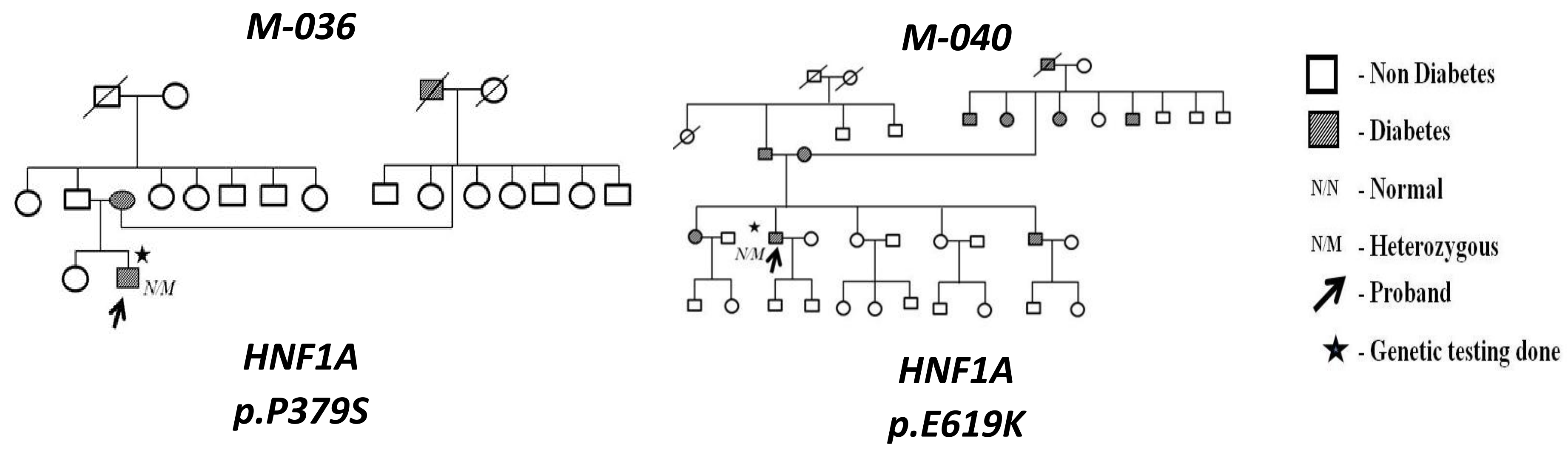

Supplementary Figure 1 : Pedigrees of MODY patients with variations in HNF1A gene (Contd…)

Pedigrees of the families showing diabetes status of each member, as well as genetic status: M/N (heterozygous), N/N (homozygous normal).

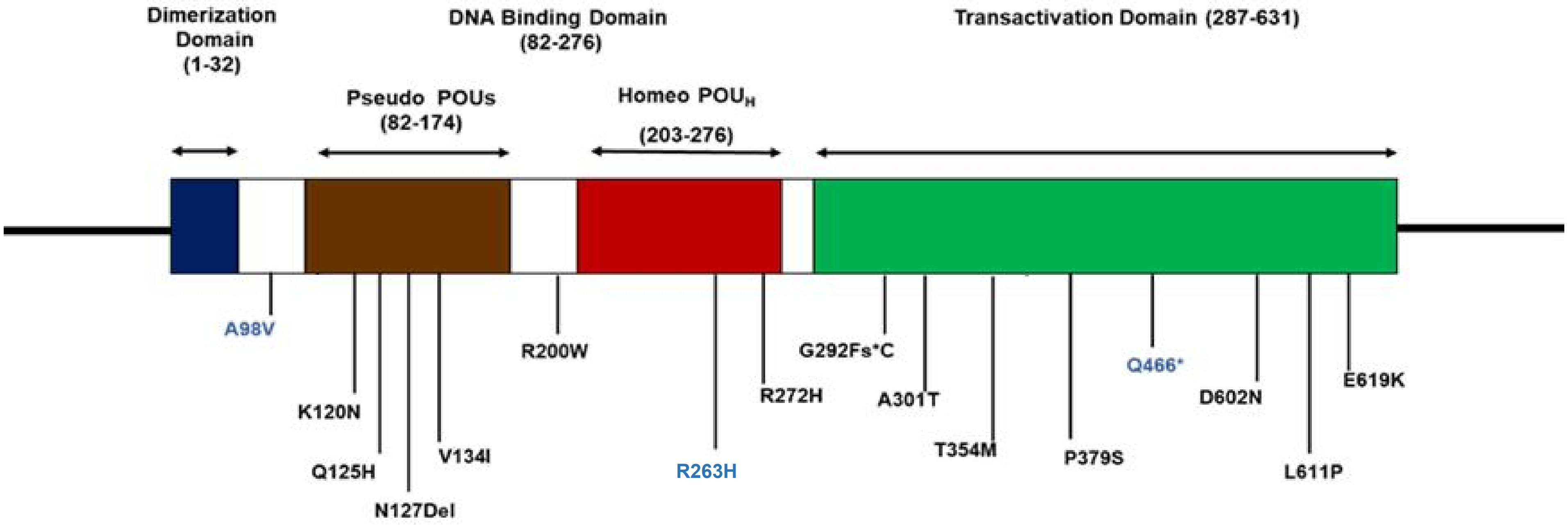

Supplementary Figure 2 : Schematic position of *HNF1A* variants in the HNF1A protein sequence identified and functionally tested in the Indian MODY subjects. Blue colour indicates the control variants used for functional analysis.

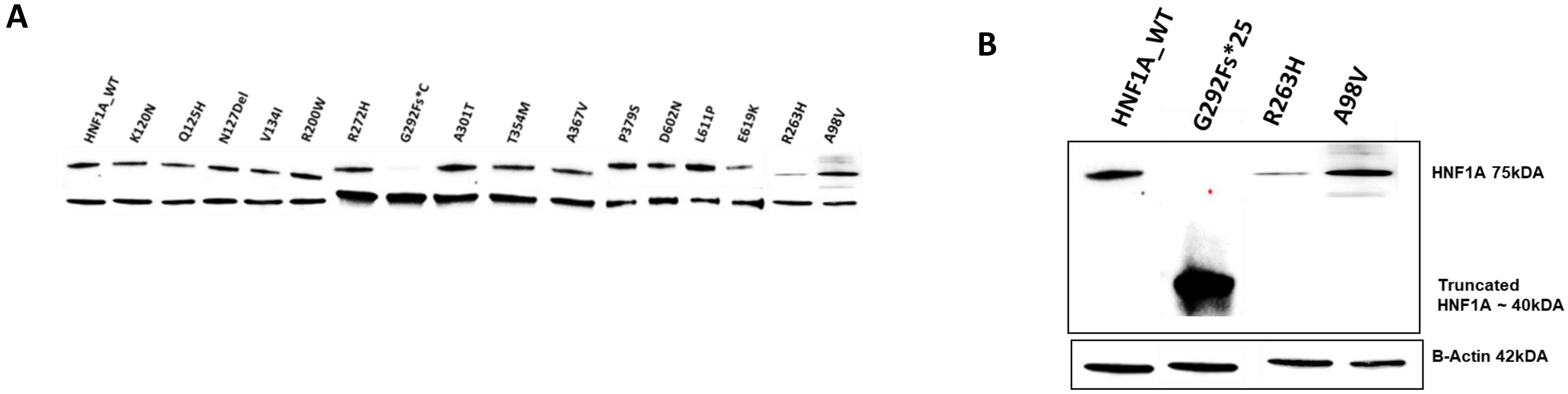

Supplementary Figure 3: **Western Blot representation of HNF1A-protein variants with B actin used as loading control.** A & B - WT or variant HNF1A was transiently transfected into HeLa cells, and the protein expression levels were analyzed by SDS-PAGE and immunoblotting using an HNF1A-specific antibody. The HNF1A protein levels were normalized to the internal loading control (actin), variant R263H analyzed in previous study was used as a control [14]. The figure is a representative image of the results obtained in 3 independent experiments.

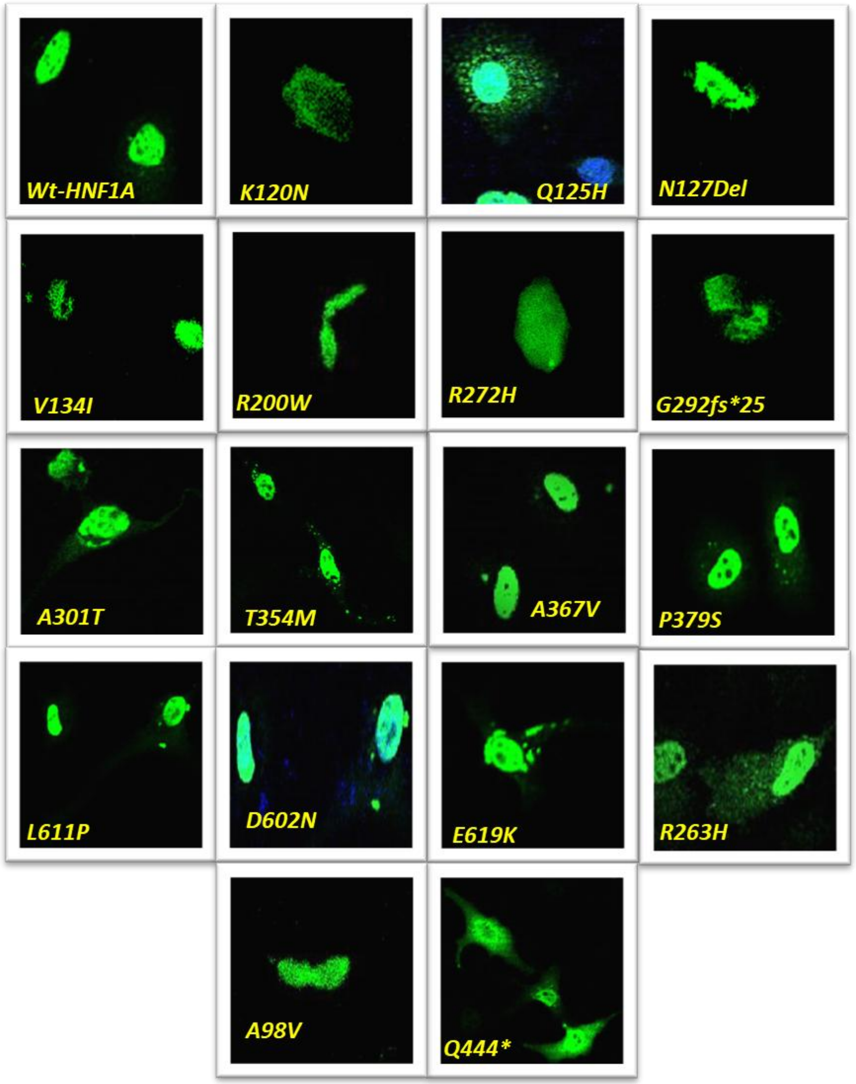

Supplementary Figure 4:***Variant effect on HNF1A protein nuclear translocation (Confocal images)***

Representative images of the nuclear localization variants in HeLa cells. One MODY3 variant (p.Q466*) with abnormal subcellular localization was included as a control. HNF1A was detected using tag-specific antibody and Alexa Fluor 488 (green). DNA staining (DAPI) is shown in blue.

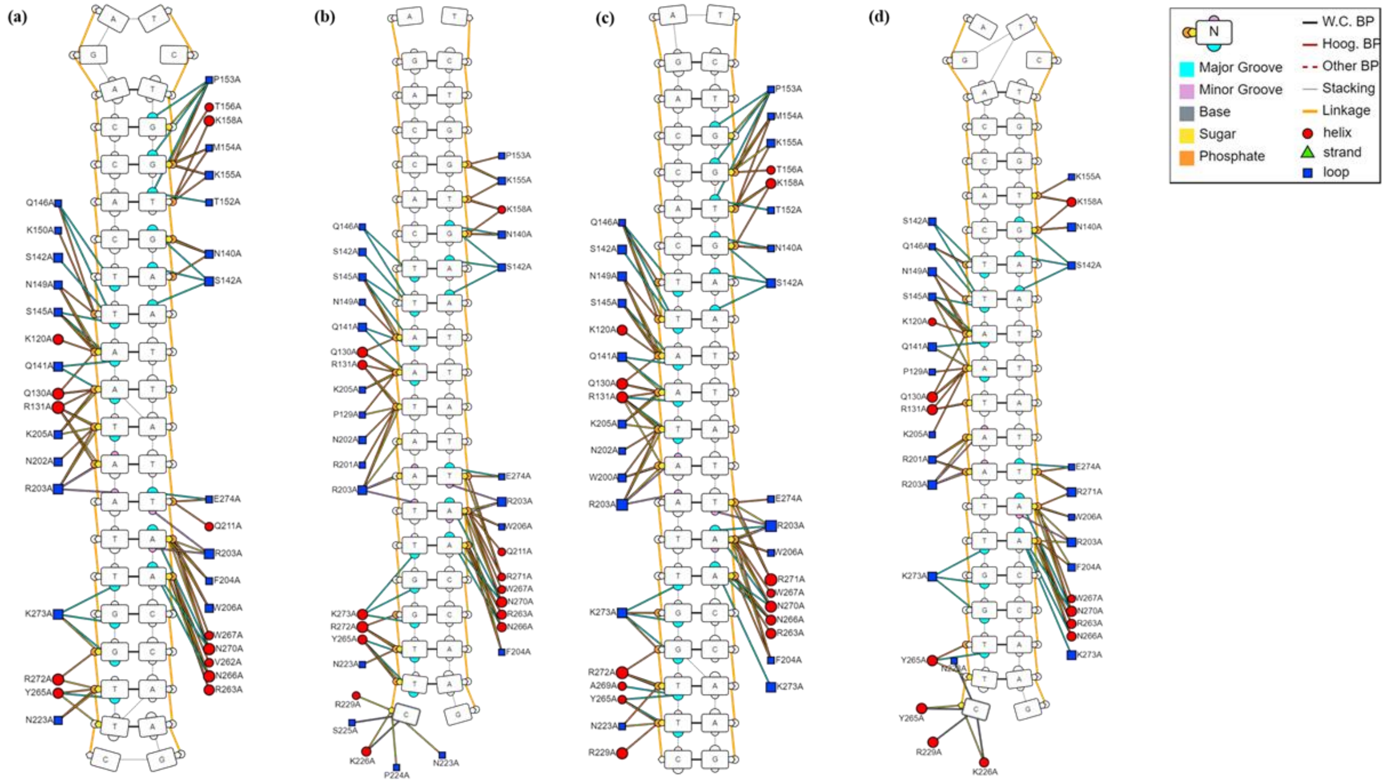

Supplementary Figure 5 : Residue Contact map.

The Residue Contact Map shows individual nucleotide-residue interactions, DNA secondary structure, protein secondary structure and DNA interaction moieties of the near-native structures of WT and MT forms of HNF1A . (a) WT HNF1A ; (b) Mutant –K120N ; (c) Mutant – R200W; (d) Mutant – R272H.The DNA is displayed as a graph, with nucleotides being nodes and edges between them indicating backbone links, base pairing or base stacking. Different base pairing geometries are indicated via the base-pair edges, and other structural features such as backbone breaks, missing phosphates, and the DNA strand sense are represented. Protein residues are displayed as small nodes with the node shape and color representing residue secondary structure. Edges between residue and nucleotide nodes represent an interaction between the two and which DNA moiety(s) the interaction involves.

2.2 Supplementary Table

Supplementary Table 1: ACMG-AMP criteria for classifying the variants in the present study

**Evidence of pathogenicity**	**Category**	
Very strong	***PVS1***	Null variant (nonsense, frameshift, canonical ±1 or 2 splice sites, initiation codon)
Strong	***PS3***	Well-established in vitro or in vivo functional studies supportive of a damaging effect on the gene or gene product (according to HGMD database [functional characterization])
Moderate	***PM1***	Located in a mutational hot spot and/or critical and well-established functional domain (e.g., active site of an enzyme) without benign variation (according to ClinVar or HGMD database)
Moderate	***PM2***	Absent from controls (according to gnomAD database [v2.1.1])
Moderate	***PM5***	Missense change at an amino acid residue where a different missense change determined to be pathogenic has been seen before (according to ClinVar or HGMD database)
Supporting	***PP3***	Multiple lines of computational evidence (using PolyPhen-2 (HumDiv), SIFT and Mutation Taster) support a deleterious effect of the missense variant
Supporting	***PP5***	Reputable source recently reports variant as pathogenic, but the evidence is not available to the laboratory to perform an independent evaluation
Supporting	***PP6***	The user has additional supporting pathogenic evidence
Strong	***BS3***	Well-established in vitro or in vivo functional studies show no damaging effect on protein function or splicing
Supporting	***BP4***	Multiple lines of computational evidence suggest no impact on gene or gene product (conservation, evolutionary, splicing impact, etc.)

Supplementary Table 2: Bioinformatic analyses of HNF1A gene variants using disease databases and prediction tools

S. No	Exon	Mutation at Protein level	Mutation at c.DNA level	Mutation Triplet codons	Chromosome Position	Databases						Prediction Analysis						
						gnomAD		ClinVar	1000G	ExAc	LOVD	Mutation Taster	PolyPhen-2	PROVEAN	SIFT	CADD Score	imutant 2.0	Grantham Score
						Allele Count	Allele Frequency											
1	2	p.Lys120Asn	c.360G>C	AAG>AAC	chr12:121426669G>C	_	_	_	_	_	_	DC	PD	Del	D	22.6	Decrease Stability	94
2	2	p.Gln125His	c.375G>C	CAG>CAC	chr12:121426684G>C	_	_	_	_	_	_	DC	B	Del	D	21.7	Decrease Stability	24
3	2	p.Asn127del	c.377_379delACA	ACA>delACA	chr12:121426686_121426688delACA	_	_	_	_	_	P	DC	_	Del	_	_	_	_
4	2	p.Val134Ile	c.400G>A	GTC>ATC	chr12:121426709G>A	1	0.000003978	_	0.0002	0.000008	_	DC	PD	Neu	D	25.9	Decrease Stability	29
5	3	p.Arg200Trp	c.598C>T	CGG>TGG	chr12:121431394C>T	_	_	Found (VCV000036824)	_	_	P	DC	PD	Del	D	23.2	Decrease Stability	101
6	4	p.Arg272His	c.815G>A	CGC>CAC	chr12:121432068G>A	_	_	Found (VCV000014931)	_	_	P, VUS	DC	PD	Del	D	29.2	Decrease Stability	29
7	4	p.Gly292fs*25	c.872-873dupC	GGG> insC	chr12:121432125_121432126dupC	_	_	Found (VCV000817605)	0.1811	0.0001375	P	DC	_	_	_	_	_	_
8	4	p.Ala301Thr	c.901G>A	GCT>ACT	chr12:121432154G>A	10	0.00004508	Found (VCV000586796)	0.0002	_	_	Poly	B	Neu	T	15	Decrease Stability	58
9	5	p.Thr354Met	c.1061C>T	ACG>ATG	chr12:121434170C>T	18	0.00006365	Found (VCV000972811)	_	0.000033	P	Poly	PD	Neu	D	21.4	Decrease Stability	81
10	5	p.Ala367Val	c.1100C>T	GCC>GTC	chr12:121434209C>T	_	_	_	_	_	_	Poly	PD	Neu	D	22.7	Increase Stability	64
11	6	p.Pro379Ser	c.1135C>T	CCT>TCT	chr12:121434371C>T	9	0.00003599	_	_	0.0069	_	DC	PD	Del	D	23.3	Decrease Stability	74
12	10	p.Asp602Asn	c.1804G>A	GAC>AAC	chr12:121438903G>A	_	_	_	_	_	_	DC	B	Neu	T	24	Decrease Stability	23
13	10	p.Leu611Pro	c.1832T>C	CTG>CCG	chr12:121438931T>C	_	_	_	_	_	_	DC	PD	Neu	D	27.5	Decrease Stability	98
14	10	p.Glu619Lys	c.1855G>A	GAG>AAG	chr12:121438954G>A	_	_	Found (VCV000972764)	_	_	P	DC	PD	Neu	D	24.9	Decrease Stability	56

P- Pathogenic; DC- Disease Causing; Poly- Polymorphism; PD- Probably Damaging; B- Benign; Del- Deleterious; Neu- Neutral; D- Damaging; T-Tolerated.

Supplementary Table 3: Clinical and biochemical workup of subjects with *HNF1A* gene variants

S. No	Patient ID	Gender	Mutation	Age at onset (Years)	Duration of Diabetes (Years)	BMI (Kg/m2)	Fasting plasma glucose (mg/dl)	Post prandial plasma glucose (mg/dl)	HbA1C (%)	Fasting C-peptide (pmol/l)	Stimulated-C-peptide (pmol/l)	Total cholesterol (mg/dl)	Triglycerides (mg/dl)	HDL (mg/dl)	LDL (mg/dl)
1	M-026	F	p.Lys120Asn	14	3.7	19.1	188	315	7.1	0.7	1.1	127	61	33	82
2	M-027	M	p.Gln125His	26	6.3	24	134	248	6.9	1	2.2	150	167	32	85
3	M-028	F	p.Asn127Del	14.9	18.1	19.1	277	414	9.5	0.6	0.8	177	134	47	101
4	M-124	M	p.Val134Ile	26.7	6.3	21.9	194	390	9.8	0.5	0.8	136	174	27	94
5	M-125	M	p.Arg200Trp	22.8	16.1	17.9	161	280	8.3	0.5	1.2	152	84	47	88
6	M-126	F	p.Arg200Trp	11	1	23.2	114	171	-	0.9	-	-	-	-	-
7	M-129	F	p.Arg272His	26	8	26.9	106	204	6.4	1.2	2	250	71	45	49
8	M-130	F	p.Arg272His	23	5	23	125	220	6.9	1	2.3	191	209	28	121
9	M-131	F	p.Gly292fs*25	19.1	13	17.3	204	197	10.8	1.1	2	211	176	44	132
10	M-035	F	p.Gly292fs*25	11	4	18.6	127	225	8.7	0.9	1.5	153	114	59	98
11	M-132	M	p.Ala301Thr	28	19	-	114	155	7.3	-	-	193	136	47	125
12	M-133	M	p.Thr354Met	24.8	5	16.2	159	243	6.9	0.7	1.3	125	77	39	71
13	M-138	F	p.Ala367Val	11.6	5	24.1	219	291	11	1	1.6	138	65	43	82
14	M-134	M	p.Pro379Ser	26	6.8	24	268	310	11.4	-	-	270	150	31	209
15	M-135	F	p.Pro379Ser	23	3	26.3	250	310	11.2	2.16	-	145	95	41	85
16	M-036	M	p.Pro379Ser	24	10	27.6	305	521	15.4	0.2	0.3	187	439	37	40
17	M-136	F	p.Pro379Ser	14	-	21.2	289	431	12.7	0.56	1.31	145	95	41	85
18	M-139	F	p.Asp602Asn	14	5	20	159	280	9	2	2.6	195	110	40	70
19	M-137	M	p.Leu611Pro	28.8	18.2	31.6	108	147	6	1.1	3	154	95	30	105
20	M-040	M	p.Glu619Lys	32	27	26.3	134	191	9.5	0.7	1.4	117	160	25	60
